# Comparative sequencing study of mismatch repair and homology‐directed repair genes in endometrial cancer and breast cancer patients from Kazakhstan

**DOI:** 10.1002/ijc.35215

**Published:** 2024-10-14

**Authors:** Ying Zheng, Natalia Vdovichenko, Peter Schürmann, Dhanya Ramachandran, Robert Geffers, Lisa‐Marie Speith, Natalia Bogdanova, Julia Enßen, Natalia Dubrowinskaja, Tatyana Yugay, Zura Berkutovna Yessimsiitova, Nurzhan Turmanov, Peter Hillemanns, Thilo Dörk

**Affiliations:** ^1^ Gynaecology Research Unit, Hannover Medical School Hannover Germany; ^2^ Genome Analytics, Helmholtz Center for Infection Research Braunschweig Germany; ^3^ Rahat Clinics Almaty Kazakhstan; ^4^ Department of Biodiversity and Bioresources al‐Farabi Kazakh National University Almaty Kazakhstan

**Keywords:** breast cancer, endometrial carcinoma, homologous recombination, mismatch repair, targeted sequencing

## Abstract

Endometrial cancer has been associated with pathogenic variants in mismatch repair (MMR) genes, especially in the context of the hereditary Lynch Syndrome. More recently, pathogenic variants in genes of homology‐directed repair (HDR) have also been suggested to contribute to a subset of endometrial cancers. In the present hospital‐based study, we investigated the relative distribution of pathogenic MMR or HDR gene variants in a series of 342 endometrial cancer patients from the Oncology Clinic in Almaty, Kazakhstan. In comparison, we also sequenced 178 breast cancer patients from the same population with the same gene panel. Identified variants were classified according to ClinVar, ESM1b, and AlphaMissense prediction tools. We found 10 endometrial cancer patients (2.9%) carrying pathogenic or likely pathogenic variants in MMR genes (7 *MSH6*, 1 *MSH2*, 2 *MUTYH*), while 14 endometrial cancer patients (4.1%) carried pathogenic variants in HDR genes (4 *BRCA2*, 3 *BRCA1*, 3 *FANCM*, 2 *SLX4*, 1 *BARD1*, 1 *BRIP1*). In the breast cancer series, we found 8 carriers (4.5%) of pathogenic or likely pathogenic variants in MMR genes (2 *MSH2*, 2 *MSH6*, 4 *MUTYH*) while 12 patients (6.7%) harbored pathogenic or likely pathogenic HDR gene variants (5 *BRCA1*, 3 *BRCA2*, 1 *BRIP1*, 1 *ERRC4*, 1 *FANCM*, 1 *SLX4*). One patient who developed breast cancer first and endometrial cancer later carried a novel frameshift variant in *MSH6*. Our results indicate that MMR and HDR gene variants with predicted pathogenicity occur at substantial frequencies in both breast and endometrial cancer patients from the Kazakh population.

## Graphical abstract

What's new?

In high‐risk families, endometrial cancer appears to be mainly triggered by pathogenic variants in mismatch repair genes, and breast cancer by pathogenic variants in homology‐directed repair genes. However, the two pathways are not mutually exclusive. This comparative single‐center sequencing study of hereditary cancer susceptibility variants in 342 endometrial cancer patients and 178 breast cancer patients in Kazakhstan indicates that gene variants from the two pathways with predicted pathogenicity occur at substantial frequencies in both breast and endometrial cancer. The broader presence of the gene variants could provide additional therapeutic opportunities for individualized treatment regimens in endometrial and breast cancer.

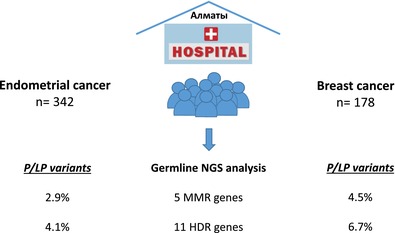

## 1 INTRODUCTION

Endometrial cancer (EC) is the sixth most common cancer in women, with a lifetime risk of ~3%.^1^ There are two main subtypes of EC, endometrioid (type I) and serous tumors (type II), with the latter being more aggressive, harder to treat, and frequently diagnosed in older women.^2^, ^3^, ^4^ While higher levels of estrogen, obesity, and higher BMI are known risk factors for type I tumors, parity, smoking status, and the use of oral contraceptives increases the risk of developing either tumor type.^5^, ^6^ There is also a known genetic risk component to EC, as seen in twin and family‐based studies.^7^, ^8^, ^9^ Microsatellite instability is frequently observed and has been associated with pathogenic variants in DNA mismatch repair (MMR) genes, such as *MSH2*, *MLH1*, *MSH6*, *PMS2*.^10^ These genes are also involved in Lynch Syndrome (hereditary nonpolyposis colorectal cancer). Patients diagnosed with Lynch syndrome and Cowden syndrome (part of the *PTEN* hamartoma tumor syndrome) are known to have an increased predisposition to developing EC.^6^, ^11^ Lynch syndrome is caused by genetic or structural variants or hypermethylation in DNA MMR genes *MLH1*, *MSH2*, *MSH6*, and *PMS2* and confers an increased predisposition to colorectal and endometrial carcinomas,^11^, ^12^ with the lifetime cumulative risk of EC for women with Lynch syndrome being in the range of 40%–60%.^13^ The lifetime risk of ovarian cancer in Lynch Syndrome may be in the range of 10%.^14^ Variants in *MSH6* and *PMS2* have been implicated in breast cancer (BC) as well^15^ but the contribution of Lynch Syndrome genes to BC has remained controversial.^12^

BC also has a strong genetic component that can give rise to familial clustering. Hereditary Breast and Ovarian Cancer (HBOC) patients are predisposed to developing BC mainly due to germline variants in DNA double‐strand break repair genes, such as *BRCA1*, *BRCA2*, *PALB2*, *BARD1*, *ATM*, *CHEK2*, *TP53*, among others.^16^, ^17^, ^18^, ^19^, ^20^, ^21^ Several of these risk genes encode proteins involved in homology‐directed repair (HDR). HDR can act on DNA double‐strand breaks to achieve non‐mutagenic repair via homologous recombination, break‐induced replication or single‐strand annealing. In these modes of repair, the single‐stranded 3′ tail generated after recognition and trimming of the break invades a homologous, intact double‐stranded DNA, forming a displacement loop. The following repair process requires DNA synthesis and ligation. Pathogenic variants of several HDR genes, including *BRCA1*, *BRCA2*, and *PALB2* have been associated with Fanconi anemia in the biallelic state or with HBOC in the mono‐allelic state.^22^

It is less clear whether pathogenic HDR gene variants also predispose to EC. A high frequency of tumors with HDR deficiency has been reported in EC (34.4%), although some half of them also harbor MMR deficiency which may have led to a secondary increase in tumor mutational burden.^22^ Initial analyses of BRCA1 carriers from HBOC families of the Breast Cancer Linkage Analysis Consortium had also suggested an increase in uterine cancer risk.^23^ However, the relationship between *BRCA1* and EC has been complicated by the fact that tamoxifen, prescribed for BC treatment, is known to increase the risk of developing type I EC,^24^ and it is currently unclear whether HDR gene variants constitute tamoxifen‐independent risk factors. However, somatic mutations in non‐endometrioid EC lead to frequent homologous recombination deficiency (HRD) due to mutations in the genes responsible for HDR.^25^, ^26^ Additionally, two recent studies found evidence for pathogenic germline *BRCA1* and *BRCA2* variants in EC cohorts.^27^, ^28^

In this study, we investigate endometrial or BC patients from the Republic of Kazakhstan for hereditary cancer susceptibility variants. This population consists of persons with Altaic ethnicities or Russian descent, with about one third of the population belonging to the latter group. According to Globocan 2020, the age‐standard incidence rate for BC is 36.3, and for EC it is 9.7 per 100,000 person‐years.^29^ A recent sequencing study has reported pathogenic variants in 12 BC predisposition genes in 22.3% of 224 Kazakh patients selected for early‐onset BC,^30^ while neither MMR gene variants nor EC cases have been assessed in this population, to our best knowledge.

Here, we performed a targeted sequencing study of the coding regions of 16 genes involved in HDR (*BARD1*, *BRCA1*, *BRCA2*, *BRIP1*, *ERCC4*, *FANCM*, *PALB2*, *RAD51B*, *RAD51C*, *RAD51D*, *SLX4*) or MMR (*MLH1*, *MSH2*, *MSH6*, *PMS2*, and the related *MUTYH*), respectively, and compared the mutational spectra between Kazakh patients with EC and Kazakh patients with BC from the same center.

## 2 MATERIALS AND METHODS

### 2.1 Patient cohorts

Peripheral EDTA blood samples were collected from a total of 472 EC patients and 279 BC patients from the region of Almaty in Kazakhstan. Patients were enrolled in this study between the years 2001–2010 and belonged to Asian (mainly Altaic) or European (mainly Russian) ancestry. These patients were unselected for age or family history or other clinical criteria, but they needed to consent and have an endometrial or breast carcinoma. Patients with non‐epithelial tumors were excluded.

In the EC series, 65% were of Russian descent, 17% were native Kazakhs, 4% Uyghurs, 4% Ukrainians, 2% Tatars, 2% Koreans, and 8% belonged to other ethnic minorities. Median age at diagnosis was 60 years (range 32–84 years) for EC. For patients with documented information on grade, 21% tumors were G1, 71% were G2, and 8% were G3. Clear cell carcinoma was present in 3% of all patients. Family history was positive in 8% of the EC patients, with 5% having a first‐degree relative and 3% having a second‐degree relative with uterine cancer.

In the BC series, 41% were of Russian descent, 35% were native Kazakhs, 6% Uyghurs, 3% Tatars, 3% Koreans, 2% Aseris, and 10% belonged to other ethnic minorities. Median age at diagnosis was 52 years (range 27–91 years). In total, 99% of the patients had invasive carcinoma. Ductal carcinoma was the main histology and accounted for 80% of the tumors. Family history was positive in 14% of the BC patients, with 5% having a first‐degree relative and 9% having a second‐degree relative with BC.

Genomic DNA was extracted from the blood samples of these patients at Hannover Medical School using standard phenol chloroform extraction, and the DNA concentration was measured using Nanodrop spectrophotometer 8000 (PeqLab). Only samples with >80 ng DNA/μL were forwarded to access array multiplex amplification and next‐generation sequencing. Based on DNA amount and quality, a total of 376 EC samples and 188 BC samples were included into the sequencing study. At further inspection of histology records, we later excluded nine EC patients and one BC patient who had sarcoma or other non‐epithelial malignancies.

### 2.2 Next‐generation sequencing

Targeted next‐generation sequencing (NGS) was performed on patients' genomic DNA samples. The regions of interest were exons and flanking intronic regions covering 5 genes from the MMR pathway (*MLH1*, *MSH2*, *MSH6*, *MUTYH*, *PMS2*) and 11 genes belonging to the homologous recombination pathway (*BARD1*, *BRCA1*, *BRCA2*, *BRIP1*, *ERCC4*, *FANCM*, *PALB2*, *RAD51B*, *RAD51C*, *RAD51D*, *SLX4*). Target‐specific primers for access array amplification were designed using the Fluidigm design platform (www.d3.fluidigm.com) with targeted amplicon size up to 300 bp. We had over 90% coverage for all attempted genes (Primer sequences in Table S1). A multiplex PCR was performed using 100–200 ng/μL DNA template, primers, and individual sample‐specific barcodes on a thermocycler as per manufacturer's instructions (Fluidigm Protocol Number 101‐7886 B2) and was loaded on the LP 48.48 integrated fluidic circuit access array (Fluidigm, USA). PCR products were pooled, cleaned up to remove PCR primers and purified by using AMPure XP magnetic beads. Illumina sequencing‐specific adaptors were added to create the sequencing libraries. All generated PCR products were checked for consistency on a 2% agarose gel before generating the amplicon pools. Each pooled library was sequenced on an Illumina MiSeq 500 system (Illumina, USA). Paired‐end sequencing was performed using the MiSeq Reagent Kit (Illumina, USA). Sequencing quality was checked by the Q30 scores and samples with scores of more than 85% were considered successful. The sequencing coverage and quality statistics for each sample are summarized in Table S2. In total, sequencing results for 342 EC patients and 178 BC patients were transferred to the further analysis.

FASTQ files obtained from the sequencing were analyzed using NextGENe software version 4.2.2 (SoftGenetics, USA). Each FASTQ file was aligned with gene‐specific .gbk files (human GrCh37). Alignment requirements and filters were as follows: matching requirements ≥80% within >30 bases; mutation percentage >25%, variant allele counts >5, total coverage counts >15.

### 2.3 Variant annotation

Known genetic variants were annotated automatically by NextGENe v.4.2.2 software (using the NCBI SNP database). Mutation taster (www.mutationtaster.org) was used to annotate any further missense or in‐frame variant from VCF files per sample. A frequency filter of MAF <0.005 was applied to remove polymorphic variants and pseudogene variants (for *PMS2*). A final table containing truncating, splice‐site, and missense variants was generated from which candidates predicted to be pathogenic in ClinVar or variants of uncertain significance were selected for validation through Sanger sequencing (see below) and taken for further analysis.

Sanger sequencing confirmed variants that were listed as pathogenic in ClinVar or that were not listed in ClinVar but generated a truncating codon before the last exon were classified as pathogenic. Variants of uncertain significance in ClinVar were further assessed using two recent prediction tools: (a) the ESM1b webtool that uses a deep‐learning protein language model trained on ClinVar to predict the degree of pathogenicity for all missense variants in the human genome (accessible at https://huggingface.co/spaces/ntranoslab/esm_variants)^31^ and (b) the AlphaMissense tool that uses structural predictions through AlphaFold to determine the effects of amino acid changes from population frequency data, structural context, and evolutionary conservation.^32^ Variants with both an ESM1b score smaller than −10 and with a prediction of pathogenicity from AlphaMissense were classified as likely pathogenic while variants with benign predictions in both ESM1b and AlphaMissense were considered likely benign. Variants with divergent predictions in the two tools (or no good prediction possibility in case of indels) were listed as ambiguous. Only pathogenic and likely pathogenic variants were taken for statistical comparisons between the EC and BC groups.

### 2.4 Sanger sequencing

Identified variants were validated in the corresponding samples by traditional Sanger sequencing using fluorescence‐labeled dideoxyribonucleoside triphosphate terminator chemistry. Primer sequences are available in Table S3. PCR amplification was performed using HotStar *Taq* DNA polymerase (Qiagen, Germany) at primer‐specific annealing temperatures. PCR products were purified by polyethylene glycol (PEG) precipitation, and the sequencing was performed using Big Dye Terminator Kit v1.1. Sequencing products were separated by capillary gel electrophoresis on a SeqStudio machine (Applied Biosystems, USA). Electropherograms were generated using the Sequencing Analysis software (Applied Biosystems, USA) and further visualized using Finch TV (Geospiza Inc., www.digitalworldbiology.com/FinchTV).

### 2.5 Statistical analysis

Statistical comparison of groups was performed using Fisher's exact test after summing up all pathogenic variants within each class of genes (HDR, MMR) per cohort. Ages at diagnosis were compared using a median test in STATA17 that is based on a continuity‐corrected Pearson χ^2^ test. The *p*‐values were two‐sided, and *α* = .05 was taken as the threshold for evidence of significance.

## 3 RESULTS

We obtained targeted germline sequencing data for 342 endometrial carcinoma patients from the Republic of Kazakhstan in the MMR genes *MLH1*, *MSH2*, *MSH6*, *PMS2*, and in the MMR‐related base‐excision repair gene *MUTYH*. Five patients were identified as harboring truncating variants: four in *MSH6* and one in *MSH2* (Table 1A). In addition, three patients harbored missense variants listed as pathogenic in ClinVar: one in *MSH6*, and two in *MUTYH* (Table 1A). Of note, one of them was compound heterozygous for two *MUTYH* variants (p.Y151C and p.G368D). We also noted rare missense variants of uncertain significance in ClinVar, with two *MSH6* variants predicted to be pathogenic by both ESM1b and AlphaMissense (Table 1A). Hence, the total number of carriers of pathogenic and likely pathogenic MMR gene variants was 10/342 (2.9%).

**TABLE 1A:** Pathogenic variants in mismatch repair (MMR) genes in Kazakh endometrial cancer patients.

Gene	Chrom. pos. (GRCh37)	Nucleotide variation	Change in mRNA	Change in protein	Molecular consequence	Variant ID	No. of patients
Pathogenic truncating variants							
*MSH2*	2:47637247–47637248	NC_000002.11:g.47637247TCTCTCTC>TCTCTC	NM_000251.3:c.387_388del	NP_000242.1:p.Gln130fs	Frameshift	rs63750924	1
*MSH6*	2:48027029–48027030	NC_000002.11:g.48027029CTCTC>TC	NM_000179.3:c.1910_1911del	NP_000170.1:p.Leu637fs	Frameshift	rs1669391378	1
*MSH6*	2:48018100–48018110	NC_000002.11:g.48018100AAGATGGAGGG>TGG	NM_000179.3:c.295_305del8*	NP_000170.1:p.Lys99fs	Frameshift	Not listed	1
*MSH6*	2:48027886	NC_000002.11:g.48027886C>T	NM_000179.3:c.2764C>T	NP_000170.1:p.Arg922Ter	Nonsense	rs587779246	1
*MSH6*	2:48026251–48026255	NC_000002.11:g.48026252AAGAGAAGAGA>AAGAGA	NM_000179.3:c.1135_1139del	NP_000170.1:p.Arg378_Arg379insTer	Nonsense	rs267608077	1
Pathogenic missense variants							
*MSH6*	2:48027436	NC_000002.11:g.48027436C>T	NM_000179.3:c.2314C>T	NP_000170.1:p.Arg772Trp	Missense	rs63750138	1
*MUTYH*	1:45798475	NC_000001.10:g.45798475T>C	NM_001048174.2:c.452A>G	NP_001041639.1:p.Tyr151Cys	Missense	rs34612342	2
*MUTYH*	1:45797228	NC_000001.10:g.45797228C>T	NM_001048174.2:c.1103G>A	NP_001041639.1:p.Gly368Asp	Missense	rs36053993	1
Variants of uncertain significance							
Likely pathogenic							
*MSH6*	2:48027221	NC_000002.11:g.48027221T>C	NM_000179.3:c.2099T>C	NP_000170.1:p.Leu700Pro	Missense	rs2104388654	1
*MSH6*	2:48027676–48027678	NC_000002.11:g.48027677AAGAAGAAGA>AAGA	NM_000179.3:c.2555del	NP_000170.1:p.Lys854del	In‐frame deletion	rs587782858	1
Ambiguous							
*PMS2*	7:6038863	NC_000007.13:g.6038863A>G	NM_000535.7:c.581T>C	NP_000526.2:p.Ile194Thr	Missense	rs2128801534	1
Likely benign							
*MLH1*	3:37090507	NC_000003.11:g.37090507A>G	NM_000249.4:c.2102A>G	NP_000240.1:p.Gln701Arg	Missense	rs2148512343	1
*MSH2*	2:47690257	NC_000002.11:g.47690257A>T	NM_000251.3:c.1474A>T	NP_000242.1:p.Met492Leu	Missense	rs774419666	1
*MSH2*	2:47709938	NC_000002.11:g.47709938G>T	NM_000251.3:c.2655G>T	NP_000242.1:p.Gln885His	Missense	rs1057521018	1
*MUTYH*	1:45798269	NC_000001.10:g.45798269T>C	NM_001048174.2:c.583A>G	NP_001041639.1:p.Ile195Val	Missense	rs200872702	1

*Note*: Variants of five sequenced MMR genes detected in 342 endometrial cancer patients are listed by chromosomal position (genome build 37), their names at the genomic, mRNA and protein level, and their variant ID in the NCBI SNP database. Variants were grouped as pathogenic truncating variants, pathogenic missense variants or variants of uncertain significance according to the ClinVar database. Variants of uncertain significance in ClinVar were grouped by their ESM1b and AlphaMissense scores are likely pathogenic, ambiguous or likely benign, respectively. Variants classified as benign in ClinVar were not considered.

We investigated the same samples in parallel in 11 HDR genes (*BARD1*, *BRCA1*, *BRCA2*, *BRIP1*, *ERCC4*, *FANCM*, *PALB2*, *RAD51B*, *RAD51C*, *RAD51D*, *SLX4*). Here, we identified 10 patients with truncating variants: 3 in *BRCA2*, 3 in *FANCM*, 2 in *SLX4*, 1 in *BRCA1*, and 1 in *BARD1* (Table 1B). An additional novel frameshift variant in *BRCA2* affected the last exon and was classified as being of uncertain significance/probably benign, this patient also carried the truncating variant p.Arg658Ter in *FANCM* (Table 1B). We furthermore identified four carriers of pathogenic missense variants: 2 in *BRCA1*, 1 in *BRIP1*, and one with an in‐frame‐deletion in *BRCA2*. Further eight variants with uncertain significance in ClinVar had ambiguous predictions or were predicted as benign. Thus, the total number of carriers of pathogenic HDR gene variants was 14/342 (4.1%).

**TABLE 1B:** Pathogenic variants in homology‐directed repair (HDR) genes in Kazakh endometrial cancer patients.

Gene	Chrom. pos. (GRCh37)	Nucleotide variation	Change in mRNA	Change in protein	Molecular consequence	Variant ID	No. of patients
Pathogenic truncating variants							
*BARD1*	2:215593433–215593434	NC_000002.11:g.215593433CACACA>CA	NM_000465.4:c.2300_2301del	NP_000456.2:p.Val767fs	Frameshift	rs750413473	1
*BRCA1*	17:41209079–41209080	NC_000017.10:g.41209078_41209079insG	NM_007294.4:c.5266dupC	NP_009225.1:p.Gln1756fs	Frameshift	rs80357906	1
*BRCA2*	13:32930691–32930692	NC_000013.10:g.32930692TCTCTCT>TCTCT	NM_000059.4:c.7567_7568delCT	NP_000050.3:p.Leu2523fs	Nonsense	rs80359664	1
*BRCA2*	13:32950854	NC_000013.10:g.32950854C>T	NM_000059.4:c.8680C>T	NP_000050.3:p.Gln2894Ter	Nonsense	rs397508002	1
*BRCA2*	13:32968950	NC_000013.10:g.32968950G>A	NM_000059.4:c.9381G>A	NP_000050.3:p.Trp3127Ter	Nonsense	rs876661242	1
*FANCM*	14:45636336	NC_000014.8:g.45636336C>T	NM_020937.4:c.1972C>T	NP_065988.1:p.Arg658Ter	Nonsense	rs368728266	2
*FANCM*	14:45667921	NC_000014.8:g.45667921C>T	NM_020937.4:c.5791C>T	NP_065988.1:p.Arg1931Ter	Nonsense	rs144567652	1
*SLX4*	16:3633255	NC_000016.9:g.3633255G>A	NM_032444.4:c.4996C>T	NP_115820.2:p.Arg1666Ter	Nonsense	rs1291935778	1
*SLX4*	16:3644579	NC_000016.9:g.3644579delG	NM_032444.4:c.2035delC	NP_115820.2:pAla679fs	Frameshift	Not listed	1
Pathogenic missense or in‐frame variants							
*BRCA1*	17:41209091	NC_000017.10:g.41209091G>T	NM_007294.4:c.5255C>A	NP_009225.1:p.Ala1752Glu	Missense	rs80357028	1
*BRCA1*	17:41267755	NC_000017.10:g.41267755T>C	NM_007294.4:c.122A>G	NP_009225.1:p.His41Arg	Missense	rs8035276	1
*BRCA2*	13:32929410–13:32929412	NC_000013.10:g.32929410GAAGAAGAA>GAAGAA	NM_000059.4:c.7420del3	NP_000050.3:p.Glu2476del	In‐frame deletion	rs876659222	1
*BRIP1*	17:59821931	NC_000017.10:g.59821931G>A	NM_032043.3:c.2119C>T	NP_114432.2:p.Arg707Cys	Missense	rs764803896	1
Variants of uncertain significance							
Ambiguous							
*ERCC4*	16:14020561	NC_000016.9:g.14020561G>T	NM_005236.3:c.532G>T	NP_005227.1:p.Val178Leu	Missense	rs149927607	1
*ERCC4*	16:14041848	NC_000016.9:g.14041848C>T	NM_005236.3:c.2395C>T	NP_005227.1:p.Arg799Trp	Missense	rs121913049	2
*PALB2*	16:23614908	NC_000016.9:g.23614908C>G	NM_024675.3:c.3433G>C	NP_078951.2:p.Gly1145Arg	Missense	rs180177137	1
Likely benign							
*BRCA2*	13:32972745	NC_000013.10:g.32972745_32972746delCinsGAATTATATCT	NM_000059.3:c.10095_10096delins	NP_000050.2:p.Ser3366fs	Frameshift (last exon)	Not listed	1
*BRIP1*	17:59876546	NC_000017.10:g.59876546G>A	NM_032043.3:c.1255C>T	NP_114432.2:p.Arg419Trp	Missense	rs150624408	1
*BRIP1*	17:59886018	NC_000017.10:g.59886018A>G	NM_032043.3:c.728T>C	NP_114432.2:p.Ile243Thr	Missense	rs587781860	1
*FANCM*	14:45668020–45668022	NC_000014.8:g.45668020GTGGTG>GTG	NM_020937.4:c.5890del3	NP_065988.1:p.Val1965del	In‐frame deletion	rs769106934	1
*SLX4*	16:3640054–3640056	NC_000016.9:3640054delAAT	NM_032444.4:c.3583_3585del3	NP_115820.2:p.Ile1195del	In‐frame deletion	rs199897550	1

*Note*: Variants of 11 sequenced HDR genes detected in 342 endometrial cancer patients are listed by chromosomal position (genome build 37), their names at the genomic, mRNA and protein level, and their variant ID in the NCBI SNP database. Variants were grouped as pathogenic truncating variants, pathogenic missense variants or in‐frame deletions, or variants of uncertain significance according to the ClinVar database. Variants of uncertain significance in ClinVar were grouped by their ESM1b and AlphaMissense scores as likely pathogenic, ambiguous or likely benign, respectively (no variant was predicted likely pathogenic in this group). Variants classified as benign in ClinVar were not considered.

We comparatively sequenced 178 BC patients from the same population and hospital with the same gene panel. The only truncating variant of an MMR gene was observed in one patient who was heterozygous for a novel frameshift deletion of 8 nucleotides within an 11‐nucleotide stretch of *MSH6* (AAGATGGAGGG>TGG, Table 2A). This patient appeared in both case series (see Table 1A) since she had been diagnosed with BC by age 50 and with EC by age 54. Her genotype and extended cancer history was not due to compound heterozygosity for two adjacent *MSH6* variants, as allele‐specific cleavage of the PCR product followed by sequencing of the uncut product showed the complex deletion in a quasi‐homozygous state, confirming a mono‐allelic mutational event (Figure S1). Three other BC patients were heterozygotes for a pathogenic missense variant in *MUTYH* (Table 2A) and, interestingly, another four missense variants with pathogenic classification from both ESM1b and AlphaMissense prediction were found in *MSH2* (*n* = 2), *MSH6* and *MUTYH* (Table 2A). By comparison, as given in Table 2B, 10 patients were identified with a truncating variant in an HDR gene (5 *BRCA1*, 3 *BRCA2*, 1 *SLX4*, 1 *ERCC4*). ESM1b and AlphaMissense furthermore predicted BRIP1*p.R823S and the novel FANCM*p.S1935I substitution, with the latter identified in two patients, as likely pathogenic. As one of the two FANCM*p.S1935I carriers also harbored the frameshift variant in *SLX4*, she was counted only once. In summary, sequencing in the BC series revealed a total of 12 carriers of pathogenic or likely pathogenic HDR gene variants (6.7%) and 8 carriers of pathogenic or likely pathogenic MMR gene variants (4.5%).

**TABLE 2A:** Pathogenic variants in mismatch repair (MMR) genes in Kazakh breast cancer patients.

Gene	Chrom. pos. (GRCh37)	Nucleotide variation	Change in mRNA	Change in protein	Molecular consequence	Variant ID	No. of patients
Pathogenic truncating variant							
*MSH6*	2:48018100–48018110	NC_000002.11:g.48018100AAGATGGAGGG>TGG	NM_000179.3:c.295_305del8 *	NP_000170.1:p.Lys99fs	Frameshift	Not listed	1
Pathogenic missense variants							
*MUTYH*	1:45798117	NC_000001.10:45798117C>T	NM_001048174.2:c.650G>A	NP_001041639.1:p.Arg217His	Missense	rs140342925	1
*MUTYH*	1:45798130	NC_000001.10:45798130G>A	NM_001048174.2:c.637C>T	NP_001041639.1:p.Arg213Trp	Missense	rs34126013	1
*MUTYH*	1:45798475	NC_000001.10:45798475T>C	NM_001048174.2:c.452A>G	NP_001041639.1:p.Tyr151Cys	Missense	rs34612342	1
Variants of uncertain significance							
Likely pathogenic							
*MSH2*	2:47672760	NC_000002.11:g.47672760T>G	NM_000251.3:c.1350T>G	NP_000242.1:p.Phe450Leu	Missense	rs2103757425	1
*MSH2*	2:47703586	NC_000002.11:47703586C>T	NM_000251.3:c.2086C>T	NP_000242.1:p.Pro696Ser	Missense	rs546201898	1
*MSH6*	2:48026567	NC_000002.11:48026567G>A	NM_000179.3:c.1445G>A	NP_000170.1:p.Arg482Gln	Missense	rs773226008	1
*MUTYH*	1:45798821	NC_000001.10:g.45798821A>G	NM_001048174.2:c.326T>C	NP_001041639.1:p.Leu109Pro	Missense	rs2149165922	1
Likely benign							
*MLH1*	3:37045908	NC_000003.11:37045908G>C	NM_000249.4:c.323G>C	NP_000240.1:p.Ser108Thr	Missense	rs1553641270	1
*MLH1*	3:37067317	NC_000003.11:7067317G>A	NM_000249.4:c.1228G>A	NP_000240.1:p.Ala410Thr	Missense	rs2083423130	1
*MSH2*	2:47705589	NC_000002.11:47705589G>A	NM_000251.3:c.2389G>A	NP_000242.1:p.Val797Ile	Missense	rs2104407230	1
*MSH6*	2:48025810	NC_000002.11:g.48025810G>A	NM_000179.3:c.688G>A	NP_000170.1:p.Glu230Lys	Missense	rs2104291160	1
*MSH6*	2:48025973	NC_000002.11:g.48025973A>T	NM_000179.3:c.851A>T	NP_000170.1:p.Asp284Val	Missense	rs1285950430	1
*MSH6*	2:48027530	NC_000002.11:48027530A>G	NM_000179.3:c.2408A>G	NP_000170.1:p.Asp803Gly	Missense	rs63751450	2

*Note*: Variants of five sequenced MMR genes detected in 178 breast cancer patients are listed by chromosomal position (genome build 37), their names at the genomic, mRNA and protein level, and their variant ID in the NCBI SNP database. Variants were grouped as pathogenic truncating variants, pathogenic missense variants or variants of uncertain significance according to the ClinVar database. Missense variants of uncertain significance in ClinVar were grouped by their ESM1b and AlphaMissense scores are likely pathogenic, ambiguous or likely benign, respectively. Variants classified as benign in ClinVar were not considered.

**TABLE 2B:** Pathogenic variants in homology‐directed repair (HDR) genes in Kazakh breast cancer patients.

Gene	Chrom. pos. (GRCh37)	Nucleotide variation	Change in mRNA	Change in protein	Molecular consequence	Variant ID	No. of patients
Pathogenic truncating or splice variants							
*BRCA1*	17:41203136	NC_000017.10:g.41203136delT	NM_007294.4:c.5278‐2delA	NP_009225.1:p.Phe1761AsnfsX14, p.Ile1760GlyfsX67	Aberrant splicing	rs878853285	1
*BRCA1*	17:41234420	NC_000017.10:g.41234420C>T	NM_007294.4:c.4357+1G>A	NP_009225.1:p.Arg1397TyrfsX2	Aberrant splicing	rs80358027	1
*BRCA1*	17:41234588	NC_000017.10:g.41234588insTCAGGTTATGTTGCATGGTATCC	NM_007294.4:c.4190ins23	NP_009225.1:p.Arg1397fs	Frameshift	Not listed	2
*BRCA1*	17:41245531–41245555	NC_000017.10:g.41245531delCTTTACCTTCCATGAGTTGTAGGTT	NM_007294.4:c.2017_2041del25	NP_009225.1:p.Glu673fs	Frameshift	Not listed	1
*BRCA2*	13:32911903	NC_000013.10:g.32911903insT	NM_000059.4:c.3410_3411insT	NP_000050.3:p.Gln1138fs	Frameshift	Not listed	1
*BRCA2*	13:32921034	NC_000013.10:g.32921034G>A	NM_000059.4:c.7007+1G>A	NP_000050.3:p.Arg2336fs	Aberrant splicing	rs397507891	1
*BRCA2*	13:32930691–32930692	NC_000013.10:g.32930691TCTCTCT>TCTCT	NM_000059.4:c.7567_7568delCT	NP_000050.3:p.Leu2523fs	Frameshift	rs80359664	1
*ERCC4*	16:14029165	NC_000016.9:g.14029165C>A	NM_005236.3:c.1376C>A	NP_005227.1:p.Ser459Ter	Nonsense	rs201179693	1
*SLX4*	16:3639302	NC_000016.9:g.3639302delGTCCG	NM_032444.4:c.4337_4341del5	NP_115820.2:p.Thr1446fs	Frameshift	Not listed	1
Variants of uncertain significance							
Likely pathogenic							
*BRIP1*	17:59793335	NC_000017.10:59793335C>A	NM_032043.3:c.2469G>T	NP_114432.2:p.Arg823Ser	Missense	rs587780239	1
*FANCM*	14:45667934	NC_000014.8:g.45667934G>T	NM_020937.2:c.5804G>T	NP_065988.1:p.Ser1935Ile	Missense	Not listed	2
Ambiguous							
*BRCA2*	13:32914792–32914797	NC_000013.10:32914792AAATGTA>A	NM_000059.4:c.6301_6306del3	NP_000050.3:p.Asn2101_Val2102del	In‐frame‐deletion	rs1593908185	1
*BRIP1*	17:59878660	NC_000017.10:g.59878660T>C	NM_032043.3:c.1094T>C	NP_114432.2:p.Ile365Thr	Missense	Not listed	1
*FANCM*	14:45669137	NC_000014.8:g.45669137T>A	NM_020937.2:c.6073T>A	NP_065988.1:p.Tyr2025Asn	Missense	rs758236096	2

*Note*: Variants of 11 sequenced HDR genes detected in 178 breast cancer patients are listed by chromosomal position (genome build 37), their names at the genomic, mRNA and protein level, and their variant ID in the NCBI SNP database. Variants were grouped as pathogenic truncating variants, pathogenic missense variants or in‐frame deletions, or variants of uncertain significance according to the ClinVar database. Variants of uncertain significance in ClinVar were grouped by their ESM1b and AlphaMissense scores as likely pathogenic, ambiguous or likely benign, respectively (no variant was predicted likely benign in this group). Variants classified as benign in ClinVar were not considered.

We investigated whether cancer patients who carried pathogenic gene variants had been diagnosed at an earlier age than noncarriers and showed a more pronounced family history. In the BC series, as expected, the eight carriers of pathogenic *BRCA1* and *BRCA2* variants were diagnosed earlier (median age 44 years, compared to 52 years in the whole series, though not statistically significant in a median test) and half of them had a first‐ or second‐degree family history of BC (compared to 13% in the whole series). No such effect was detected in the four carriers of pathogenic or likely pathogenic variants in other HDR genes including *BRIP1*, *ERCC4*, *FANCM*, or *SLX4* (median age at diagnosis 67 years, no familial cases), nor in the eight carriers of pathogenic or likely pathogenic MMR gene variants (median age at diagnosis 53.5 years, no familial cases; Table S4). In the EC series, the median age at diagnosis was 54 years (range 44–70 years) for MMR gene variant carriers, 64 years (range 50–79 years) for patients with a pathogenic HDR gene variant, and 60 years (range 32–84 years) for the whole cohort (Table S5). We did not detect gross variations in the distribution of histology subgroups, as far as they were recorded, but pathogenic HDR gene variants were associated with high grade endometrioid cancer (*p* = 0.004, 2df), driven by *BRCA1* and *BRCA2* (Table S5). However, grade was underdocumented in our series and numbers were small. First‐degree family history for EC was positive in 4/10 (40%) patients with a pathogenic MMR gene variant and in 2/14 (14%) patients with a pathogenic HDR gene variant, compared to 13/318 (4%) in noncarriers (*p* = .0009 for MMR, Fisher's exact test, 2 df ).

## 4 DISCUSSION

EC and BC are among the most common female cancers. Both of them can run in high‐risk families, with EC often associated with colorectal cancer, and BC more frequently associated with ovarian cancer. In such families, EC appears to be mainly triggered by pathogenic variants in MMR genes, and BC by pathogenic variants in HDR genes. The presence of such repair gene variants may not just increase the risk of cancer but also predict the therapeutic response toward DNA damaging treatments. It may also increase the tumor mutational burden which is thought to improve the response toward immunotherapy through generation of neoantigens. Tumor mutational burden is an FDA‐approved marker for immunotherapy response although this is controversely debated.^33^ However, it is clear that the identification of hereditary variation in DNA repair genes can have a substantial impact on family counseling and therapeutic decisions.

While there is long‐standing evidence for the differences in tissue‐specific expressivity of cancer in patients with pathogenic MMR or HDR gene variants, MMR and HDR pathways are not mutually exclusive and the repair proteins involved interact physically in DNA damage signaling networks, for example, *BRCA1* and *MLH1* or *BRIP1* and *MSH2*.^34^ It is thus possible that MMR deficiency also to some extent predisposes to BC, and HDR deficiency also to EC. Previous studies have remained inconclusive in this regard. For example, the BC Association Consortium has sequenced MMR genes in 60,466 BC cases and 53,461 controls and found a significant difference for truncating variants in *MSH6* but not for other MMR genes.^18^ Some supportive but still ambiguous evidence has come from the analysis of multiple‐case families. A study of 423 women with Lynch Syndrome revealed an increased standard incidence ratio of BC for carriers of pathogenic variants in *MSH6* or *PMS2* but not *MSH2* or *MLH1*.^15^ Vice versa, an early study of the Breast Cancer Linkage Consortium revealed an increased risk of uterine cancer in carriers of pathogenic *BRCA1* variants^23^ but this has not been replicated in subsequent studies.^35^, ^36^ In a study of EC patients selected from HBOC families, 9 out of 21 patients (42.8%) showed a pathogenic variant in *BRCA1* or *BRCA2* ^28^ but such an occurrence could be due to segregation by chance in first‐degree relatives of known mutation carriers. In a prospective cohort study of 627 carriers of a pathogenic variant in *BRCA1*, a higher than expected incidence of EC was observed.^37^ However, it has been argued that some members of HBOC families may take tamoxifen as a preventive measure that is known to increase EC risk.^38^ To overcome such biases, additional case–control studies are needed outside of HBOC families and excluding tamoxifen‐induced EC. A recent review surveyed the hitherto available studies and concluded that, despite mounting evidence indicating the EC risk in carriers of pathogenic germline *BRCA1* or *BRCA2* variants, data is still insufficient to indicate hysterectomy for all women with such variants.^39^

In this work, we compared HDR and MMR gene variants in BC and EC series from the same hospital and with the same gene panel and sequencing method. We wanted to define the landscape of mutations of these genes in Kazakh patients and to determine how much the germline mutational spectra in EC and BC differ from each other. We did not find significant differences in the mutational distribution between the two case–control series. However, our panel included several genes for which variants confer modest or unknown risks (such as *FANCM* or *MUTYH*). When the comparison was restricted to genes with variants of higher penetrance (*BRCA1*, *BRCA2*, *MSH2*, *MLH1*, *MSH6*, *PMS2*), the prevalence of *BRCA1* and *BRCA2* variants was about twofold increased in BC patients compared to EC patients (*p* = .16, 2 df) but the presence of 4 *BRCA2* and 3 *BRCA1* pathogenic variants in the EC series still appeared high (2.0%). On the other hand, Lynch Syndrome gene variants remained similar in both case series (2.3% and 2.2%, respectively).

We found 4/178 BC patients with pathogenic or likely pathogenic variants in Lynch Syndrome genes (2 *MSH2*, 2 *MSH6*). One patient who first developed BC and 4 years later EC, had entered both series and was found to carry a novel frameshift variant in *MSH6*. As tamoxifen was not part of BC therapy in Kazakhstan at the time of recruitment, the genetic predisposition may have supported the development of both cancers. Of note, missense substitutions constitute a substantial fraction of the pathogenic variants in Lynch Syndrome genes. The frequency of rare missense variants in Lynch Syndrome genes may be up to 10% in BC patients^18^ but it has been difficult to classify these variants according to their pathogenicity. The recently developed ESM1b and AlphaMissense tools provide improved classifications^31^, ^32^ and have been helpful to predict the effect of such missense variants in our study.

Our results for EC are comparable to a recent sequencing study of 307 Chinese EC patients of whom 17 (5.5%) carried a pathogenic variant in the MMR genes *MSH2*, *MSH6* and *MLH1*, whereas 6 (2.0%) carried a pathogenic variant in *BRCA1* or *BRCA2*.^40^ Another study of 527 Czech EC patients found pathogenic variants in MMR genes in 27 (5.1%) patients and *BRCA1* or *BRCA2* pathogenic variants in 18 (3.4%).^41^ Compared to those studies, we have lower frequencies of pathogenic MMR gene variants in the Kazakh EC patients. But previous study cohorts were enriched for familial cases and Lynch Syndrome which was not a selection criterion in our study. Large unselected EC series from Ohio had revealed pathogenic variants in Lynch Syndrome genes in 1.8% or 3% of cases, respectively, which comes closer to our results.^42^, ^43^ The latter study also reported 10/961 (1%) EC cases with pathogenic variants in *BRCA1* or *BRCA2*, and 9/961 cases (0.9%) with pathogenic variants in other HDR genes including *PALB2*, *BRIP1*, and *RAD51C*.^43^ Differences in population frequencies, different gene panels and methodological differences may limit the comparability between studies. However, they would not affect the comparison of distinct cancer series from the same population within the same study as provided here.

Our results allowed a direct comparison of patients with breast or EC from the same large hospital center, and it also extends our knowledge about the mutational spectrum of MMR or HDR genes in the Republic of Kazakhstan. When this work had started, initial reports of *BRCA1* and *BRCA2* mutation analyses in Kazakh cancer patients had provided anecdotal evidence^44^ or did not detect pathogenic variants.^45^ Meanwhile, a larger study using gene panel sequencing has indicated a significant occurrence of pathogenic variants in BC predisposing genes, including *BRCA1* and *BRCA2*, in 224 Kazakh patients with early‐onset BC.^30^ It is likely from these and our sequencing data that HDR and MMR gene variants occur at similar frequency in Kazakh and in European patients. Although the previous BC study reported some variants at a higher frequency,^30^ no such variant stood out in our unselected series. Among previously unreported variants, only *BRCA1* variant c.4190ins23 and *FANCM* variant p.Ser1935Ile were observed in two unrelated patients, suggesting an absence of large founder effects for specific variants in the ethnically heterogeneous population of Kazakhstan. We noticed that Russians constituted the majority of patients in both series, followed by Kazakhs. This may reflect the known two‐ to three‐fold higher cancer incidence rates in Russians compared to Altaic and East Asian populations. The Russian proportion was higher in the EC series compared to the BC series (62% vs. 42%). However, we don't think that a genetic difference has significantly influenced our overall comparison of HDR and MMR variants because we did not find a particularly common founder mutation in the two series. Furthermore, the HDR:MMR ratio for pathogenic gene variants was the same in Russians as it was in the total series (1.4 in EC, 3 in BC; Table S6). The higher impact of *BRCA1* and *BRCA2* among the HDR genes and of *MSH6* among the MMR genes, and the relatively high abundance of recessive missense variants in *MUTYH* are also in line with findings from other populations. *SLX4* is less known than the others, because its pathogenic variants are rare, although one large sequencing study suggested a potential role for ovarian cancer risk.^46^ In our study, this gene was found mutated in three patients, including two with EC, suggesting that a screening of *SLX4* in larger series may be warranted. Limitations of our study include the lack of sequencing data for the healthy population of Kazakhstan and the absence of extended immunohistochemical or HRD profiles for more precise tumor characterization.

While first‐degree family history of EC was 10‐fold enriched for carriers of MMR gene variants, our study was not able to clarify a potentially increased family history of uterine cancer in close relatives of HDR variant carriers, which would have implications for genetic counseling and surveillance. There was also no evidence for an earlier age at diagnosis of EC in HDR variant carriers, contrasting with some previous case reports.^47^, ^48^ Larger studies will be needed to clarify the impact of HDR gene variants on EC risk. However, half of the identified pathogenic HDR gene variants in EC patients were in *BRCA1* or *BRCA2*, which calls for counseling in regard of breast and ovarian cancer risk in some 2% of EC patients and their family members from Kazakhstan. Interestingly, the *BRCA1* and *BRCA2* variants also were associated with high grade of endometrial carcinoma in the identified carriers. As the numbers were small, this observation needs to be followed in much larger studies.

There are clinical implications of the relatively high frequency of HDR gene variants in regard that standard therapy in EC is based on taxanes and platinum. Pathogenic variants in *BRCA1* or *BRCA2*, or an HRD signature, are associated with favorable outcome of platinum therapy in other cancers such as ovarian cancer,^49^, ^50^, ^51^, ^52^ triple‐negative BC,^53^ pancreatic cancer,^54^ or lung cancer.^55^ It is unknown whether a better therapeutic response of carriers of high‐penetrance variants in *BRCA1* or *BRCA2* is similarly observed for EC. HDR inactivation may occur in every sixth endometrial tumor,^56^ and initial evidence suggests that disease‐free survival may be worse in patients with wild‐type HDR genes.^57^ Additional treatments, such as PARP inhibitors with or without anti‐PD‐1/PD‐L1 therapy, could also become an option for EC patients with deficiency of homologous recombinational repair. Interestingly, PARP inhibition has been reported to be potentially effective in EC tumors and is currently under investigation in clinical trials.^58^ An assessment of HRD scores in the tumor could supplement selection of patients for platinum or PARP inhibitor therapy.^59^ This may be relevant for patients with pathogenic variants in either HDR or MMR genes, because MMR‐deficient tumors have a higher likelihood for biallelic inactivation of HDR genes through somatic mutation.^60^, ^61^ Germline predisposition such as those identified in our study would increase this likelihood further.

In summary, this study has compared the germline mutational spectra of selected MMR and HDR genes in EC and BC patients from the same center and population in Kazakhstan. We find both groups of genes are mutated in a significant fraction of both cancer series, consistent with the view that their tissue specificity might be broader than anticipated. The presence of MMR gene variants in BC and HDR gene variants in EC could provide additional therapeutic opportunities for individualized treatment regimens.

## AUTHOR CONTRIBUTIONS

**Ying Zheng:** Investigation; methodology; validation; writing—original draft; writing—review and editing. **Natalia Vdovichenko:** Investigation; methodology; validation; writing—review and editing. **Peter Schürmann:** Investigation; methodology; validation; writing—review and editing. **Dhanya Ramachandran:** Formal analysis; investigation; validation; writing–original draft; writing—review and editing. **Robert Geffers:** Formal analysis; investigation; validation; writing—review and editing. **Lisa‐Marie Speith:** Investigation; writing—review and editing. **Natalia Bogdanova:** Conceptualization; supervision; writing—review and editing. **Julia Enßen:** Data curation; investigation; writing—review and editing. **Natalia Dubrowinskaja:** Conceptualization; data curation; writing—review and editing. **Tatyana Yugay:** Conceptualization; data curation; resources; writing—review and editing. **Zura Berkutovna Yessimsiitova:** Conceptualization; data curation; resources; writing—review and editing. **Nurzhan Turmanov:** Conceptualization; data curation; resources; supervision; writing—review and editing. **Peter Hillemanns:** Funding acquisition; supervision; writing—review and editing. **Thilo Dörk:** Conceptualization; data curation; formal analysis; funding acquisition; project administration; supervision; writing—original draft; writing—review and editing.

## FUNDING INFORMATION

Y.Z. received a stipend from Wu'an First People's Hospital. T.D. and P.H. received funding from the Wilhelm Sander Foundation (Grant no. 2021.142.1).

## CONFLICT OF INTEREST STATEMENT

The authors declare that the research was conducted in the absence of any commercial or financial relationships that could be construed as a potential conflict of interest.

## ETHICS STATEMENT

The study received ethical approval from the institutional review board at Hannover Medical School (vote 5833), and informed consent was obtained from the patients at the clinics in Almaty.

## Supporting information


**Data S1:** Supporting Information.

**Table S1:** Primer sequences for MMR and HDR gene amplification.

**Table S2:** Sequence coverage and quality statistics per sample.

## Data Availability

Genomic coordinates and primer sequences are available as Supplementary Files S1. Other data that support the findings of this study are available from the corresponding author upon request.
