# Supplementary material for: Comparative sequencing study of mismatch repair and homology‐directed repair genes in endometrial cancer and breast cancer patients from Kazakhstan
Source: Int J Cancer. 2024 Oct 14;156(4):764–75. doi: 10.1002/ijc.35215 (PMC11661514; doi:10.1002/ijc.35215)
Supplement: Supplementary file 1 — Data S1: Supporting Information. [file IJC-156-764-s002.pdf]

Comparative sequencing study of mismatch repair and homology-directed repair genes in endometrial cancer and breast cancer patients from Kazakhstan

Ying Zheng, Natalia Vdovichenko, Peter Schürmann, Dhanya Ramachandran, Robert Geffers, Lisa-Marie Speith, Natalia Bogdanova, Julia Enßen, Natalia Dubrowinskaja, Tatyana Yugay, Zura Berkutovna Yessimsiitova, Nurzhan Turmanov, Peter Hillemanns, Thilo Dörk

List of content:

Supplementary Figure S1: Mono-allelic sequencing of *MSH6* variant c.295_305del8.

Sanger sequencing of two four-nucleotide deletions of AAGA and AGGG in a patient with breast and endometrial cancer (bottom panel) compared to a wildtype reference sample (upper panel). This figure shows the sequencing after treatment of the PCR product with *MnlI* that selectively cleaves the reference sequence. The residual mutant sequence clearly showed that both four-nucleotide deletions occur on the same strand, thus establishing phase and indicating a novel frameshift deletion of 8 nucleotides within an 11-nucleotide stretch of *MSH6* (AAGATGGAGGG>TGG).

Supplementary Table S1 (uploaded as separate excel file): Primers and amplicons for MMR and HDR genes.

Primer sequences and the amplicon sizes and chromosomal positions are provided for targeted PCR amplification using multiplex assays on Fluidigm access arrays (design by Standard BioTools).

Supplementary Table S2 (uploaded as separate excel file)**:** Sequence coverage and quality statistics per sample.

Coverage is provided per sample as median and mean together with range and first and third quartile values. We included percentage of targeted bases with coverage >=15, and also provide the percentage of targeted bases with coverage >=200 (outmost right columns).

Supplementary Table S3: Primers for Sanger validation of MMR and HDR gene variants.

Primer sequences are provided for targeted PCR amplification of selected regions harboring NGS-detected variants of the indicated genes for their subsequent validation through Sanger sequencing.

Supplementary Table S4: Clinical features of endometrial cancer in carriers of pathogenic HDR or MMR variants.

Statistical comparison with non-carriers was performed using a Pearson-based median test (p_{median}) or Fisher´s exact test (p_{exact}), respectively. pV HDR, pathogenic variant in a homology directed repair gene; pV MMR, pathogenic variant in a mismatch repair gene. Non-carriers (neither pV HDR nor pV MMR) were taken as the reference group.

Supplementary Table S5: Clinical features of breast cancer in carriers of pathogenic HDR or MMR variants.

Statistical comparison with non-carriers was performed using a Pearson-based median test (p_{median}) or Fisher´s exact test (p_{exact}), respectively. pV HDR, pathogenic variant in a homology directed repair gene; pV MMR, pathogenic variant in a mismatch repair gene. Non-carriers (neither pV HDR nor pV MMR) were taken as the reference group.

Supplementary Table S6: Ethnicity distribution among carriers of HDR or MMR gene variants.

Numbers and percentages are given for three main ethnic subgroups (Russians, Kazakhs, Uygurs) and others. The latter included in the endometrial cases (*) Ukrainians (13), Koreans (10), Tatars (9), Germans (6), Azerbaijanis (2), Dungans (2), Chechen (1), Polish (1), Armenian (1), Ashkenazi (1), Belarusian (1), Czech (1), unknown (3), and in the breast cancer cases (**) Koreans (8), Azerbaijanis (6), Tatars (4), Turks (2), Kurds (2), Ukrainians (2), Germans (2), Uzbek (1), Chechen (1), Polish (1), Dungan (1), Udmurt (1), Armenian (1), Balkar (1), Lezgin (1). HDR, homology directed repair; MMR, mismatch repair; pV, pathogenic variant.

Supplementary Figure 1: Identification of *MSH6* variant c.295_305del8

*MSH6**wildtype

*MSH6**p.Lys99fs
(monoallelic
after *Mnl*I treatment)

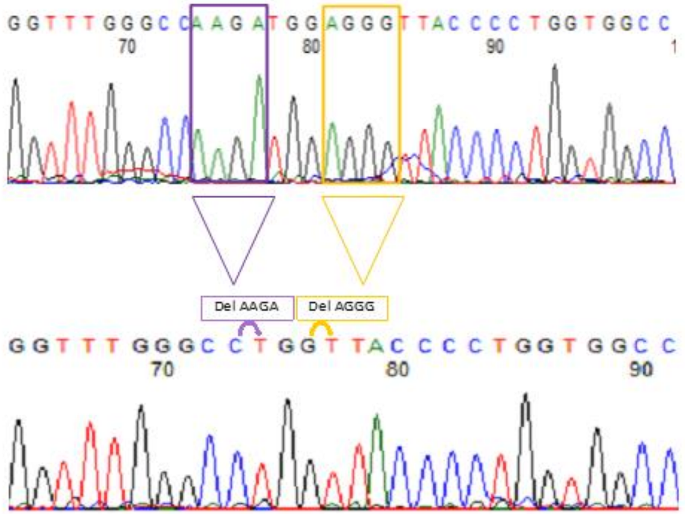

Supplementary Table S3: PCR primers for selected MMR and HDR gene regions to validate variants through Sanger sequencing

Gene	Chr.	Pos.	Forward Primer (5´-3´)	Reverse Primer (5´-3´)
BARD1	2	215593433	ATTCTGATCAGCGCTTCTG	GTACAATGACTGGGCTCTC
BRCA1	17	41267755	ACTCAGTCATAACAGCTCAAAG	GTTAGGTGTTTCCTGGGTTATG
BRCA1	17	41209079	TCTCTTATCCTGATGGGTTGTG	GGAGTGGAATACAGAGTGGTG
BRCA1	17	41245531	GGAAAGTATCGCTGTCATGTC	TCTACCAGGCATATTCATGCG
BRCA1	17	41234420	AGAGCTATGTTGTATACCATG	AACTTGTAGTTCCATACTAGG
BRCA1	17	41234588	CTTATGATGGAAGGGTAGCTG	GGACATCACAATAACATCAAG
BRCA1	17	41203136	CTAGAACATTTCAGCAATCTG	ACATTGGACTGATTGTCCCTG
BRCA2	13	32930691	CAACGCGTCTTTCCACAG	AGAACACGCAGAGGGAAC
BRCA2	13	32950854	CCTTCTTTGGGTGTTTTATGC	GTCTGCTGCATTCTTCACTG
BRCA2	13	32929410	CATTGATGGACATGGCTCTG	CGGAAATATCTAACTGAAAGGC
BRCA2	13	32968950	GCCTCATATGTTAATTGCTGC	TGGACTAGCAGAAAACACAG
BRCA2	13	32972745	CTGACGAAGAACTTGCATTG	CTCACATTCTTCCGTACTGG
BRCA2	13	32921034	TGAGCATCTGTTACATTCACTG	AAACGAGACTTTTCTCATACTG
BRCA2	13	32914792	CTGCTATACGTACTCCAGAAC	GAGTTTACACAGTGCTCTGG
BRCA2	13	32911903	CTGCACATTTACAGAGTAGTG	GAGCTTCACTAGAAACATTCAG
BRIP1	17	59821931	TACTTGCCGTAGTCACATTG	CTCCCTGTGGTTCTACAATG
BRIP1	17	59886018	CACTGTTCTAGGTGCTGTTG	TACCTCAGGATGGACACAAG
BRIP1	17	59793335	GGTAATTTAAGGAATGTGAAGC	TTTAAGGCCCTGTATGCTTG
BRIP1	17	59876546	AGAAGCCTAGTTAACCAAAG	ATAACATCGAGGACTGTGC
BRIP1	17	59878660	AGACTTTCCAAGGGATGTGC	ATCTCCATGAGTAGGAAGAAGG
ERCC4	16	14041848	GTGCTTCTGATTGAGTTTGACC	GGCAGTTTTTGGCATTCACC
ERCC4	16	14020561	GAGCCCACAGAATAATCGAG	CCACAGATACAGTTTCCTCAC
ERCC4	16	14029165	GAGAGACTATATCACTCTTGG	GCTACTGCTTATTTCTCGACG
FANCM	14	45668020	GGAACTGTCTTTAGTGGAAC	GACATACCTGTTAGCCATCC
FANCM	14	45636336	AGAAGTCCACGAATGGTTCC	CGACCTGCTAAGTAAGAACAG
FANCM	14	45667921	AGACACATCAAGGATGTTTAGG	TTGTTCCACTAAAGACAGTTCC
FANCM	14	45669137	GTTCAAGACATATCAGCTGGG	GCAATGATTATTGAAGTGCAT
FANCM	14	45667934	AGACACATCAAGGATGTTTAGG	TTGTTCCACTAAAGACAGTTCC
MLH1	3	37090507	TGCGCTATGTTCTATTCCATCC	GCTGACTGTTGTGTTTGAACCT
MLH1	3	37045908	AGTGGGTGACCCAGCAGTGAG	GTCAGCACTATACCTGTATGC
MLH1	3	37067317	GAAGTAGTGATAAGGTCTATG	GGCCACAGGACCATTCCAGCA

MSH2	2	47637247	AGTTGAGATCGCACCATTGC	TGTCTCTGGCCATCAACTGC
MSH2	2	47690257	GTCACTTTGTTCTGTTTGCAG	TGACAGAGATGTGAAGTCATC
MSH2	2	47709938	CAATTTGTCACTGTCTAACATG	CATTACTGGGATTTTTCACGTAG
MSH2	2	47703586	CTGAGGATAGAAGCAGTTTCC	CATCAGTGTACAGTTTAGGAC
MSH2	2	47705589	ATGTGCTTCAGGTCTGCAACC	GAGTACTCCAATAGTACATAC
MSH2	2	47672760	GACTCCTCTTACTGATCTTC	CCACAAAGGTGCTACAATTAG
MSH6	2	48027886	CATGGAAGAAGTTGCTGATGG	TCTCTAGGTATTCCAGGAGGC
MSH6	2	48027029	GAGTTCATTGTCCTGTTCTC	GTCATACCTTTAAGCACCTG
MSH6	2	48026251	GTTAGTGGAGGTGGTGATG	TCTGAGACTTAATCTGCCAC
MSH6	2	48027676	TAGAAGACCTCATGGTTGTGC	CAGCAACTTCTTCCATGATCC
MSH6	2	48027436	ATGGTGCTAGATGCAGTGAC	TCAGGCACAACCATGAGGTC
MSH6	2	48027221	AAGTGAATTGGCCCTCTCTG	ATGTCACTGCATCTAGCACC
MSH6	2	48026567	CACCTTCCAGCACACTGTAAG	CTCAGAACTTTGATCTTGTCATC
MSH6	2	48025810	ACCTATCTCAGACGTGCTCAG	GCGACTACTGTCATCACCACC
MSH6	2	48027530	CAGCAACTTCTTCCATGATCCC	GGGTTGATACTTGCCATACTCC
MSH6	2	48018100	TGCCAGAAGACTTGGAATTG	AAGCCTTTTGCTAACCCAG
MUTYH	1	45798475	TAGGAACAATAGAGGGACTG	ATTGGAGTGCAAGACTCAAG
MUTYH	1	45797228	GCATGAGTAACAAGAGAGAATG	CTATTCCGCTGCTCACTTAC
MUTYH	1	45798269	GAGTCTTGCACTCCAATCAG	AGAGGGGCCAAAGAGTTAG
MUTYH	1	45798821	CTGCATCCATCCGGTATAGTA	CATTGACAGGCAGAAGATGAG
MUTYH	1	45798117	GTCCACCAGCTGCTGGGCTAG	GCCACGTACAGCAGAGACCCT
MUTYH	1	45798130	GTCCACCAGCTGCTGGGCTAG	GCCACGTACAGCAGAGACCCT
PALB2	16	23614908	GACGTGAAAGATCACTGTGC	CAAATGAGAGTCTGTACCCG
PMS2	7	6038863	TGAGAACCTTGCGTTGGATG	AAGTAACCGGCCATCACTAC
SLX4	16	3640056	TGAGGAGCTGGAGCTAGAAC	TGCTCTCACGGTCACAGAAC
SLX4	16	3644579	TTAGGGTCACTCAGAGGTTG	ACGAACTTGTGGGCGTAAAG
SLX4	16	3633255	TCGCCTCCCAGACCTACAAG	ATCATTGAGGCCTGGAGGTG
SLX4	16	3639302	GTCCCACAGAGCCGAATTCAG	GTCGGAGACAGTGACGATGAG

Supplementary Table S4: Clinical features of endometrial cancer in carriers of pathogenic HDR or MMR variants

**Mutation status**	**Age at diagnosis**			**Histology**			**Grade**				**Firstdeg FH(EC)**		
	Range	*Mean*	*p(median)*	*Type I*	*Type II*	*p(exact)*	*1*	*2*	*3*	*p(exact)*	*Yes*	*No*	*p(exact)*
pV HDR	50-79	62.1 (57.5-66.8)	0.40	5	0	1.00	0	1	3	**0.002**	2	12	0.13
pV MMR	44-70	55.9 (50.8-61.0)	0.09	2	0	1.00	1	1	0	1.00	4	6	**0.001**
Non-carriers	35-84	60.2 (59.2-61.3)	Ref.	86	14	Ref.	23	58	5	Ref.	13	305	Ref.

Supplementary Table S5: Clinical features of breast cancer in carriers of pathogenic HDR or MMR variants

**Mutation status**	**Age at diagnosis**			**Histology**			**Firstdeg FH(BC)**		
	Range	*Mean*	*p(median)*	*Ductal*	*Lobular*	*p(exact)*	*Yes*	*No*	*p(exact)*
pV HDR	27-91	55.4 (43.7-67.0)	0.46	11	1	0.46	2	10	0.15
pV MMR	46-58	51.8 (43.7-59.8)	0.35	2	1	0.15	0	4	1.00
Non-carriers	29-72	52.2 (50.6-53.9)	Ref.	127	6	Ref.	9	169	Ref.

Supplementary Table S6: Ethnicity distribution among carriers of HDR or MMR gene variants

**Endometrial cancer**	**No. (%)**	**pV HDR**	**pV MMR**
Russian	210 (61.4%)	10	7
Kazakh	63 (18.4%)	1	2
Uyghurs	18 (5.3%)	2	1
Others*	51 (14.9%)	1	0
All endometrial cancer	342 (100 %)	14	10
**Breast cancer**	**No. (%)**	**pV HDR**	**pV MMR**
Russian	76 (42.7%)	6	2
Kazakh	58 (32.6%)	5	1
Uyghurs	10 (5.6%)	0	1
Others**	34 (19.6%)	1	0
All breast cancer	178 (100 %)	12	4
